# Inter-rater reliability of clinical mobility measures in ankylosing spondylitis

**DOI:** 10.1186/s12891-016-1242-1

**Published:** 2016-09-05

**Authors:** J. Calvo-Gutiérrez, J. L. Garrido-Castro, C. González-Navas, M. C. Castro-Villegas, R. Ortega-Castro, C. López-Medina, P. Font-Ugalde, A. Escudero-Contreras, E. Collantes-Estévez

**Affiliations:** Rheumatology Department, Reina Sofía University Hospital, Maimonides Institute for Biomedical Research of Cordoba (IMIBIC), University of Cordoba, Av/ Menedez Pidal SN, 14001 Cordoba, Spain

**Keywords:** Ankylosing spondylitis, Reliability, Reproducibility, Smallest detectable difference, BASMI

## Abstract

**Background:**

Several measurements are often used in daily clinical practice in the assessment of Ankylosing Spondylitis (AS) patients. The Assessment in SpondyloArthiritis International Society (ASAS) recommend in its core set: chest expansion modified Schöber test, Occiput to wall distance, lateral lumbar flexion, cervical rotation and The Bath Ankylosing Spondylitis Metrology Index (BASMI). BASMI also includes five measurements, some of them recommended by ASAS. Three versions of BASMI have been published with different scales and intervals for each component of the index. Though studies about reliability of these measurements are needed. The aim of this study was to analyze inter-rater reliability of recommended spinal mobility measures in AS.

**Methods:**

We examined reproducibility of spinal mobility measurements on 33 AS patients performed by two experienced rheumatologists in the same day. Descriptive statistics, Intraclass Correlation Coefficients (ICC), and Smallest Detectable Difference (SDD) using the Bland-Altman criteria were obtained for all the measurements.

**Results:**

Chest expansion showed the lowest value of ICC (0.66) and occiput-wall the highest (0.97). SDD was 2.43 units for BASMI_2_ and 1.27 units for BASMI_10_.

**Conclusions:**

Reliability according to ICC was moderate to high in all measurements. BASMI_10_, instead BASMI_2,_ must be used: measurements used to calculate are the same but there is better reliability. Inter-rater variation, expressed as SDD, must be taken in account: smaller improvements do not demonstrate the efficacy of treatment because they can be due to experimental error and not to the treatment itself.

## Background

Ankylosing Spondylitis (AS) is a subtype of Spondyloarthropaties (SpA), which affects mainly the spine. Spinal mobility impairment in AS patients is caused both by inflammation and structural damage of the spine [1, 2]. Assessment of the reduction in spinal mobility is fundamental to evaluate disease stage and disease evolution in the patients [3]. Several studies showed that the evolution of the disease is highly correlated with the reduction in spinal mobility [4, 5]. Several measurements were defined to assess spinal mobility in AS, among them due to their greater acceptance are those recommended by ASAS (The Assessment of SpondyloArthritis international Society) group its comprise different spinal mobility measures in a core set [6]: chest expansion, modified Schöber test, occiput to wall distance, lateral lumbar flexion or BASMI (Bath Ankylosing Spondylitis Metrology Index).

BASMI was defined in 1994 by Jenkinson et al. [7] and includes five measurements: cervical rotation, tragus to wall distance, lateral lumbar flexion, modified Schöber, and intermalleolar distance. Each one of these measurements is classified in three levels of severity (0 = Mild, 1 = Moderate, 2 = Severe) according to values defined for each interval. Summing up the results obtained for the five measurements, an index between zero and ten is obtained. This index was validated in several studies showing good reliability and correlation with radiological measures [8]. BASMI is used as a tool for patient’s classification and to analyze the sensitivity to change of different treatments. In 1995, a second definition of BASMI was published by Jones et al. [9], using the same measurements, but establishing ten intervals in each measurement. Averaging individual scores, an index between zero and ten is obtained. This new scale gave a greater precision of the evaluation obtained by each measurement (multiples of 0.2 units instead multiples of 1 unit). More recently, Van deer Heidje et al. [10] proposed a BASMI version in which a linear function was applied for calculate each index component. According to the authors, this definition of BASMI, whose results were very similar to Jenkinson’s version, provides better results of the reliability and sensitivity to change. Thus, there are three versions of BASMI: original BASMI_2_, BASMI_10_ and linear BASMI_LIN_.

Another metrological indexes and new measurement protocols have been defined [11, 12] but the most used spinal mobility measurements are the ones recommend by ASAS.

BASMI is not always performed in daily clinical practice, due to the difficulty to obtain certain measurements (cervical rotation with a goniometer, intermalleolar distance needs more physical space). Although BASMI_2_ is used more than BASMI_10_, the latter has higher accuracy, for the same measurements. Although in most publications BASMI_2_ is used, it is often not clear to the readers which of the three definitions were applied.

In this study, we analyzed inter-observer variability of different spinal mobility including ASAS core set and BASMI, with its three different versions. Our aim was to obtain reliability of the spinal measurements and to determine the smallest detectable difference, which must be considered in order to demonstrate the efficacy of the treatment assessed with spinal measurements.

## Methods

### Patients

We included 33 consecutive patients from daily clinical practice from Rheumatology Department of University Hospital Reina Sofia, Córdoba. Inclusion criteria were: patients diagnosed with AS according to the modified New York criteria, having at least 5 years of disease duration and with ages between 18 and 80 years. They were all informed and consented to participate in the study, who was approved by the Reina Sofia Hospital Research Ethics Committee. Exclusion criteria were: pregnant, spinal surgery and scoliosis.

Only four of them were female. These patients had different level of mobility impairment varying from 0.94 to 8.78 (average value 4.75) according to BASMI_10_. The medium age was 50.35 years, and disease evolution was 24.61 years.

Spinal measurements were performed by two experienced rheumatologists. Two assessments in independent and isolated way were performed in the same day by each rheumatologist. All tests were done in the evening during three months period.

### Variables

There are more than 20 measurements used for AS assessment. Mobility measures were reviewed by Sieper et al. [13]. This review makes a precise description of the most used metrology measures (including all the measures analyzed in our study) and how to calculate them.

ASAS recommends chest expansion, modified Schöber and occiput to wall distance and lateral lumbar flexion or BASMI. BASMI includes: cervical rotation, tragus to wall distance, lateral lumbar flexion, modified Schöber and intermalleolar distance. Three ranges of BASMI were considered: BASMI_2_, BASMI_10_, BASMI_LIN_. As, the last two are very similar we will use only BASMI_2_ and BASMI_10_. Finger to floor distance was also included because it is a measure often used in studies. In total, we analyzed eight measurements and two indexes.

### Statistics

We used intraclass correlation coefficient (ICC) for statistical analysis of inter-observer reliability. A value upper to 0.6 indicates good reliability, a value superior to 0.8 indicates a very good reliability and upper to 0.9 represents an excellent reliability. Determination of the smallest detectable difference was made using Bland-Altman method [14]. According to this method, 95 % limit of confidence is defined as the difference measured between observers for each measurement +/− 1.96 the standard deviation. Assuming that the differences are normally distributed the mean difference must be near zero. We used SPSS® 14.0 (SPSS International BV, Chicago, USA) and Medcalc® 11.3.6 (Medcalc Software bvba, Mariakerke, Belgium) to interpret the results.

## Results

Results obtained by both observers for the analyzed parameters are shown in Table 1. High variability was observed in chest expansion and Schöber test. Values over one unit in BASMI_10_ and two units in BASMI_2_ indicated high values of SDD.

**Table 1:** Results obtained by observers and differences between measurements

Measurement	Observer 1	Observer 2	Difference	C.V.	S.D.D.
Chest expansion ^a^ (cm)	3.45 (1.73) [1.50–11.00]	3.61 (2.27) [1.00–11.50]	−0.15 (1.67) [−8.50/2.00]	47.31 %	3.27
Modified Schöber ^a^ ^b^(cm)	2.79 (1.59) [0.00–6.00]	2.99 (1.90) [0.00–7.00]	−0.21 (1.22) [−3.50/3.20]	42.81 %	2.39
Occiput-wall ^a^ (cm)	7.52 (6.56) [0.00–19.00]	6.71 (6.12) [0.00–17.00]	0.80 (1.63) [−2.00/6.00]	22.91 %	3.19
Tragus-wall ^b^ (cm)	17.97 (5.96) [10.50–28.00]	17.31 (5.53) [11.00–26.00]	0.64 (1.56) [−2.00/5.00]	8.84 %	3.06
Floor to finger distance (cm)	26.05 (11.43) [5.00–50.00]	26.15 (11.75) [3.50–48.00]	−0.09 (3.57) [−12.00/8.00]	13.68 %	6.99
Lateral lumbar flexion ^a,b^ (cm)	8.30 (5.89) [1.00–26.50]	8.20 (5.16) [1.00–19.50]	0.09 (3.19) [−9.00/10.50]	38.67 %	6.25
Intermalleolar distance^b^ (cm)	92.67 (17.75) [50.00–117.00]	90.89 (17.39) [50.00–118.00]	0.97 (4.37) [−9.00/12.00]	4.76 %	8.56
BASMI_10_	4.81 (2.11) [0.94–8.74]	4.67 (2.29) [0.96–8.78]	0.14 (0.65) [−1.41/1.66]	13.71 %	1.27
BASMI_2_^a^	4.17 (2.71) [0.00–8.75]	3.97 (2.69) [0.00–8.75]	0.20 (1.24) [−2.50/3.50]	30.47 %	2.43

Results of observers and difference are expressed as mean (SD) [Min-Max]

*CV* coefficient of variation: SD of difference divided by mean value in %, *SDD* smallest detectable difference according Bland-Altman criteria

^a^ Included in ASAS core set. ^b^ Included in BASMI

Table 2 shows inter-observer reliability according to ICC. Results of reliability compared with already published studies are also included. Although ICC values are high, for a good instrument for individual decision-making, these values must be over 0.9. Not all measurements fulfill this condition.

**Table 2:** Reliability obtained in our study and published by other authors

Measurement	ICC Inter (95 % CI)	Davis [15]	Haywood [8]	Maksym.[11]	Viitanen [3]
Chest expansion ^a^ (cm)	0.659 (>0.412)	0.85	-	0.98	0.85
Modified Schöber ^a,b^ (cm)	0.756 (>0.561)	0.96	0.90	0.97	0.96
Occiput-wall ^a^ (cm)	0.967 (>0.934)	0.92	0.98		0.89
Tragus-wall ^b^ (cm)	0.962 (>0.924)	0.99	-	0.96	0.90
Finger to floor distance (cm)	0.948 (>0.893)	0.98	0.96		
Lumbar side flexion ^a, b^ (cm)	0.817 (>0.651)	0.98	0.95	0.95	0.98
Intermalleolar distance ^b^ (cm)	0.944 (>0.857)	0.99	-	0.98	
BASMI_10_	0.956 (>0.913)	-	-		
BASMI_2_^a^	0.894 (>0.796)	0.96	-	0.95	

^a^Included in ASAS core set. ^b^Included in BASMI

Correlations between measures (Pearson) are showed in Table 3. High correlation values appeared in BASMI indexes with the rest of measures.

**Table 3:** Correlation (Pearson) between measures

	CE	MS	OW	TW	FFD	FL	ID	BASMI_10_	BASMI_2_
CE	1.000	0.435*	−0.334	−0.360*	0.057	0.234	0.377	−0.448**	−0.499**
MS	0.435*	1.000	−0.409*	−0.370*	−0.275	0.510**	0.070	−0.610**	−0.667**
OW	−0.334	−0.409*	1.000	0.979**	0.262	−0.676**	−0.552*	0.817**	0.688**
TW	−0.360*	−0.370*	0.979**	1.000	0.187	−0.707**	−0.572*	0.802**	0.713**
FFD	0.057	−0.275	0.262	0.187	1.000	−0.438*	−0.563*	0.442*	0.316
FL	0.234	0.510**	−0.676**	−0.707**	−0.438*	1.000	0.366	−0.800**	−0.765**
ID	0.377	0.070	−0.552*	−0.572*	−0.563*	0.366	1.000	−0.697**	−0.520*
B_10_	−0.448**	−0.610**	0.817**	0.802**	0.442*	−0.800**	−0.697**	1.000	0.916**
B_2_	−0.449**	−0.667**	0.688**	0.713**	0.316*	−0.765**	−0.767**	0.916**	1.000

*p < 0.05; ** *p* < 0.01

*CE* chest expansion, *MS* modified Schöber, *OW* occiput to wall distance, *TW* Tragus to wall distance, *FFD* finger to floor distance, *ID* intermalleolar distance B_10_:BASMI_10_. B_2_:BASMI_2_

**Fig. 1:**
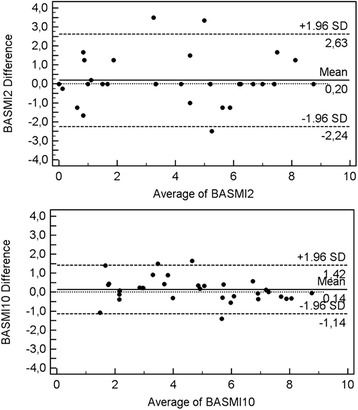
Bland-Altman plots illustrating inter-observer variability of assessing BASMI according BASMI_2_ and BASMI_10_

Figure 1 shows Bland-Altman plots comparing the scores of the two BASMI definitions obtained for both observers.

## Discussion

The main conclusion of our study is that inter-observer variability expressed as SDD must be kept in mind in order to justify patient improvement for short-term follow-up treatments. Every measurement included in ASAS core set and BASMI was analyzed.

Davis et al. [15] in a bibliographical review, studied the different spinal mobility measurements used to assess loss of mobility in AS, including BASMI, analyzing their validity applying the OMERACT filter (Outcome Measures in Rheumatoid Arthritis Clinical Trials). Although Davis shown good results, some studies show certain problems of reliability, accuracy and variability of these mobility measurements. Auleley et al. [16] calculated the smallest detectable difference (SDD) of several measurements used in AS assessment (chest expansion, occiput to wall distance and modified Schöber). This was the first study providing SDD as outcome measurement in AS based on Bland-Altman’s 95 % limits of agreement method [14]. The SDD was relatively high and, although ICCs were high, it appeared to be poorly reliable judged by SDD. Consequently, changes smaller than SDD, could be considered as measurement error.

Different reliability results were obtained, for each measures analyzed. Next we will review some of these results comparing them with the obtained in the current literature.

Chest expansion showed the worst results (CV 47.31 %, ICC 0.659, SDD 3.27 cm). Is one of the most complicated measures to be obtained and it has special problems to be done in women. According to Auleley [16] ICC was 0.85 and SDD 2.4 cm. This measurement may be useful, because the reduction of pulmonary capacity in AS is well known, but the actual measure system is not appropriate. Tzelepis et al. [17] used the thoracoabdominal movement in breathing as outcome parameter of the disease.

Modified Schöber is one of the mobility measures more used. Clear correlations with radiology and symptoms duration has been described [3]. Reliability results were good (CV 42,8 %, ICC 0.756, SDD 2.39 cm), higher than chest expansion but lower than the rest of the measures. Our results were very similar with Auleley [15] (ICC 0.60, SDD 3.3 cm).

Occiput-wall / Tragus-wall distances are related with kyphosis seen in AS. Greater level of affectation implies greater kyphosis. ASAS recommends occiput-wall, although this parameter is difficult to measure when the patient is only some millimeters separated from the wall. Measuring tragus-wall is easier in this situation (BASMI includes this measure), but this distance depends on the size of the patient’s head. In BASMI_2_, values less than 15 cm are scored with 0 (no affectation). Normally the distance tragus to occiput is about 11 cm. In this case, there is no problem if the patient touches the wall. However, in BASMI_10_, the zero value for this measure is 10 cm, so the patient will be scored with 1 unit and will be affected according this index. Some studies [18, 19] assessed BASMI in healthy subjects and they discovered that it is unusual for healthy individuals to score zero on the BASMI. Both measures, obtained good reliability results (especially tragus to wall). Also they showed good correlations with the rest of parameters. Therefore, kyphosis is a good indicator of the level of affectation.

Floor to finger distance is not included in ASAS core set neither in BASMI. In spite of having a high reproducibility (ICC = 0.948), it does not correlate well with BASMI index (*r* = 0.44), nor with the rest of parameters. This fact can be due to the influence of the height of the subject, although some studies declined it [20]. Some studies show poor correlation with radiology for this measure [21].

Unlike finger to floor distance, lateral lumbar flexion showed good correlation with the rest of measures (Schöber, occiput and tragus to wall distance, and BASMI, *p* < 0.01). ICC was good (0.817), something inferior than obtained by other authors (0.95–0.98).

Intermalleolar distance measure showed excellent values of repeatability (ICC = 0.944) and good correlations with some of the analyzed parameters. It is complex to measure and requires more space and time in clinical practice, for this reason is not habitually used.

We have calculated BASMI using the two scales previously described (BASMI_2_.and BASMI_10_). BASMI_10_ showed better results than BASMI_2_. BASMI correlated well with almost all of the measures. Floor to finger distance did not show correlation with this index. We obtained a SDD of BASMI_2_ of 2.43 units, while for BASMI_10_ it was half the value (1.27 units). This result is shown in Fig. 1. CV also varied from 30.47 to 13,71 %. ICC was good in both cases (0.894 to 0.956); therefore the variability is smaller for the second index. The published results of variability are near to the values obtained in our study.

According to Madsen [22] the SDD, in BASMI_2_ was of +/− 1.4 (+/− 2 units in valuations on individual patients). Another study performed by Martidale et al. [23] shown that for repeat assessments of the same participant, differences in BASMI of 1.0 or less are within bounds of error. Therefore, an improvement below these units may be due to the experimental error and not to the treatment itself.

Some studies showed improvements of less than one unit in BASMI_2_ and BASMI_10_ in patients treated with biological agents [24, 25]. These values are less than the smallest detectable difference established for BASMI and therefore, the improvement could be due to the experimental error of the measure. It is a fact that BASMI is seldom used to evaluate the short-term effectiveness of the treatment. Some authors prefer to use lateral side flexion instead BASMI [26]. Braun et al. [27] indicated that although biological treatment improves AS activity indices, this improvement is less important with the respect to spinal mobility (assessed with BASMI), but he strengthened out that BASMI does not have much sensitivity to change. Jauregui et al. [28] analyzed BASMI in a controlled trial using pamidronated and they concluded that responsiveness of the BASMI was poor with either scoring system (BASMI_2_ and BASMI_10_).

To summarize, BASMI_10_ must be used because the measures included are the same and requires only little extra effort in its calculation. Although we obtained better results for BASMI_10_, BASMI_2_ is still using in clinical practice.

As a limitation of our study, the results we provide for SDD are based on a relative small number of patients and observers but, the results are similar to other studies.

## Conclusions

In order to analyze clinical significance of our results, SDD of the different measurements must be kept in mind when demonstrating the efficacy of treatments in short term studies. Another possibility is to research for advanced metrology tools with better reliability results to assess mobility in AS patients.

## Abbreviations

AS: Ankylosing spondylitis
ASAS: Assessment of Spondylo Arthritis International Society
BASMI: Bath ankylosing spondylitis metrology index
CV: Coefficient of variation
ICC: Intraclass correlation coefficient
OMERACT: Outcome measures in rheumatoid arthritis clinical trials
SDD: Small detectable difference
SpA: Spondyloarthropaties
